# On the Absorption of Dead Bone, Royal Medical & Chirurgical Society, April, 1864

**Published:** 1864-11

**Authors:** Wm. Scovell Savory

**Affiliations:** Assistant-Surgeon to St. Bartholomew's Hospital.


### CHRONICLE OF MEDICAL SCIENCE.

*On the Absorption of Dead Bone.* By WM. SCOVELL SAVORY, Esq., F. R. S., Assistant-Surgeon to St. Bartholomew's Hospital. Royal Medical & Chirurgical Society, April, 1864.

Can dead bone be absorbed? This question still awaits a satisfactory answer. For while careful and accurate experiments have furnished only negative results, there are unquestionable facts which compel us to admit the possibility of the occurrence. One all-important consideration seems to have been hitherto neglected in the inquiry—the influence of pressure in determining the result. Thus, in the experiments which have been performed on the subject, and which have naturally led to the conclusion that dead bone may be kept amidst living tissues for weeks or months without losing the merest fraction of its weight—in these experiments the dead bone was kept in simple contact only with living parts. It appears that no considerable pressure was maintained. Whereas when ivory pegs are driven into bone, extreme pressure is of course produced. In order to test this view, some experiments were performed which are related in the paper. It appeared to the author that the only explanation which can be offered of the results of these several experiments is, that the absorption of dead bone, when in contact with living bone, is determined by the *pressure* to which it is subjected.

The President said the communication was interesting in connection with the experiments of Mr. Gulliver. The state of the fragments in cases of ununited fracture had been noticed before; but it had not been attributed to the cause which was explained in Mr. Savory's paper.

Mr. Hilton said the profession ought to feel obliged to Mr. Savory for having adduced, by well-considered and well-arranged experiments, such conclusive evidence of the absorption of dead bone by the surrounding living tissues—a fact not usually admitted by surgeons. He (Mr. Hilton) had several times noticed, on looking at two ivory pegs which had been employed in the same case of united fracture, and apparently under the same conditions, that the surface of one of them was partially absorbed, whilst the other did not manifest any loss of substance—a difference hitherto inexplicable, but now elucidated by the author's paper, as depending upon the variable pressure to which they had been subjected. An interesting point, however, presented itself for consideration, to which the author had not made any reference—viz: what was the amount and duration of pressure required to induce this absorption? for dead bone was often seen buried within granulations which were undoubtedly capable of exerting much pressure without the slightest appearance of any absorption having occurred. For instance, in the case of an amputation through the femur, the same end of the bone may come away necrotic after several months' subjection to the pressure of muscles, fascia, granulations, bandaging and strapping, yet the track of the teeth of the saw used at the amputation would be seen as cleanly cut and as sharply defined as on the day of the original operation. The same kind of facts was quite as discernible in cases of compound fracture of a long bone, where the fractured end of the bone, although surrounded deeply by granulations and new bone during several months, would present the sharp, well-defined edge of the fracture as evidently as on the day of the accident, uninfluenced by the pressure of any of the surrounding living tissues. Mr. Hilton had removed from the leg several portions of a comminuted compound fracture of the tibia eight years after the accident and seven years after the closure of the external wound, and upon two of them the well-defined edge of the original fracture was obvious and markedly different from the serrated edge observable where the piece of bone had been separated from the living bone by the slow process of absorption. Mr. Hilton would suggest to the author the inquiry as to how or by what combination of minute events does pressure contribute towards absorption of dead bones, because the pressure in his (Mr. Savory's) experiments was made equally on both the living and dead bone. No doubt such an investigation could not be placed in better hands than those of the author of the paper.

Mr. Partridge inquired whether the absorption of the pegs was greater in the shaft of the bone or in the medullary canal.

Mr. Savory said absorption was greater in the shaft portion than in the medullary canal.

Mr. Coote said that Mr. Savory's experiments were very interesting in confirming the view entertained by many surgeons that, in the removal of dead bone, the action of the absorbents was possible in the material that had perished. He had himself witnessed the corrosion of the surface of ivory pegs introduced into living bone for the purpose of promoting union in a non-united fracture. The possibility of the fact, which had been doubted, was important; but, after the experiments thus detailed, could not be denied. He (Mr. Coote) thought that perhaps pressure alone was not sufficient to explain the phenomena of removal. He had witnessed the separation of large portions of bone, which he believed to be dead, on which no pressure could have been exerted. But the fact remained the same; and the value of Mr. Savory's observations must be undisputed. He (Mr. Coote) concluded with some remarks on the relative size of sequestra and the bony cavities which contained them.

Mr. Brooke said that he doubted whether the process by which the surface of the pegs was eroded could truly be designated a process of absorption. He thought it probably analogous to the process of disintegration, by which the surface of a sequestrum is acted on by the highly vascular granulating surface with which it is contact. That disintegration, and not absorption, is going on in cases of necrosis, may generally be rendered evident by the appearance of the *debris* of bone-tissue in the pus discharged from the cloaca.

Mr. Wood asked the author whether, in each of the experiments detailed, the condition of the medullary membrane in contact with the peg had been observed, as regarded the presence of granulations or other absorbing structure. In many of the cases in which the pegs had been loosely inserted, extensive suppuration, and in some instances death, had followed. Could the action set up in these cases have been that of a process destructive to the absorbing or vital powers of the bone and medullary membrane operated on, this would of course prevent the occurrence in these experiments of any absorption of the foreign or dead bone. He presumed that in all the experiments pegs made of the same dried bone had been used, and that there was in no case introduction of putrid matter. In most of the pegs shown in the box handed round which exhibited any evidence of roughening, this extended equally through the parts embedded in the bone of the animal, although only the part traversing the compact tissue of the cylinder of bone had been subject to pressure. The intermediate portion had been in contact with the medullary substance. In the specimen from which the drawing was taken the peg seemed even more attenuated in the cavity of the bone than where in contact with its walls.

Mr Solly remarked that, while agreeing with all the former speakers in the great value of Mr. Savory's paper, he did not think that it subverted all the old opinions that dead bone could not be absorbed except under pressure: for instance, in necrosis of the parietal bones—a good case of which he had in his mind's eye—where the bone died, became black, lost its sensibility, and was then separated from the living bone because it was dead. The sequestrum which was thus cast off was perforated in all directions as though worm-eaten, and absorbed in patches. Was not this an instance of dead bone being absorbed without pressure? Had not every surgeon in the room seen such cases?

Mr. Savory, in reply, said he had considered it best in the paper simply to demonstrate the fact that the absorption of dead bone is determined by the pressure to which it is subjected. In working at the matter, of course he had thought of the nature of the influence thus exerciseed, but he did not consider any opinion which he might have formed on the subject worth expressing. The question was not in relation to the absorption of the bone, whether living or dead, but to the effect of pressure on the absorption of *dead* bone. With respect to the case Mr. Solly mentioned, it was not enough to show that dead portions of bone bore evidence of having been partially absorbed: it must be shown that such absorption occurred after the death of the bone, and thus independently of all pressure. Mr. Savory defended the use of the word "absorption." He had not employed the term without foreseeing the objection that might be urged against it; and so he had been careful to relate how, in some of the experiments, the wounds at once closed and completely healed without any discharge or other means by which disintegrated fragments of bone might have escaped. Moreover, if the preparations were examined, it would be seen that, in some of them, the portions of dead bone which had been removed could not have escaped, for the holes were tightly plugged by the pegs which had been driven in. With reference to the destination of the bone which disappears in disease, Mr. Savory thought that the evidence advanced to prove that this is always disintegrated and cast out, was unsatisfactory and inconclusive. Of course in some forms of ulceration of bone, as in phagedenic ulcers of soft parts, disintegrating fragments might perish and escape; but in other less destructive forms of ulceration bone might disappear through absorption. Much had been made of the fact that the discharge from carious bone contains an unusual abundance of phosphate of lime, this being supposed to represent the dissolved osseous tissue. But while, on the one hand, this would prove too much, the proportion of bone which disappears not being equal to the quantity of phosphate of lime discharged; on the other hand, a better, a more philosophical explanation of the fact might be given. As in health each part assimilates to itself from the blood its own proper constituents, so in abnormal forms of nutrition it was reasonable to believe that the material furnished by different structures would present characters of composition more or less corresponding with those of the tissue whence it proceeded. Be this as it might, however, in some at least of the experiments described there was no means by which the portion of bone which had disappeared could have escaped externally.

